# Chromosome 11q23 aberrations activating *FOXR1* in B-cell lymphoma

**DOI:** 10.1038/bcj.2016.43

**Published:** 2016-06-10

**Authors:** C Pommerenke, V Hauer, M Zaborski, R A F MacLeod, S Nagel, R M Amini, M Berglund, R Geffers, H G Drexler, H Quentmeier

**Affiliations:** 1 Leibniz-Institute DSMZ-German Collection of Microorganisms and Cell Cultures, Braunschweig, Germany; 2 Department of Immunology, Genetics and Pathology, Uppsala University, Uppsala University Hospital, Uppsala, Sweden; 3 Genome Analytics Research Group, Helmholtz Centre for Infection Research, Braunschweig, Germany

Recurrent chromosome 11q23 abnormalities, including focal gains and losses have been described in mantle cell lymphoma, diffuse large B-cell lymphoma (DLBCL) and in a subset of high-grade B-cell lymphomas lacking *MYC* rearrangements.^1, 2, 3^ We describe a novel fusion of *FOXR1* forkhead box gene, located at 11q23, with a neighboring gene in B-cell lymphoma.

RNAseq and sequencing of cloned PCR products revealed fusion transcripts of 5′ *Ribosomal Protein S25* (*RPS25*) with *FOXR1* in the DLBCL cell line U-2932, both genes located at the amplified chromosomal region 11q23 (Figure 1a, Supplementary Figure S1A). Genomic cloning localized the breakpoint to intron 2/3 of *RPS25* and to the promoter region of *FOXR1* (bp −3532).

Cell line U-2932 comprises two distinct clones traceable to subclones present in the patient's tumor.^4^ These differences also affected 11q32, *FOXR1* and *RPS25* being tetraploid in subclone R1, and triploid in subclone R2 (3n) (Supplementary Figure S1A). In accordance with the genomic data, the *RPS25*/*FOXR1* fusion was detected in subclone R1 but not in R2 (Figure 1b). *RPS25*/*FOXR1* was also verified in the patient's DNA, which collectively suggested that the fusion had occurred at some later stages of tumor development (Figure 1b).

Physiological *FOXR1* (formerly *FOXN5*) expression is restricted to the early stages of embryogenesis.^5, 6^ Ectopic expression as result of 11q23 intrachromosomal deletion-fusion has hitherto been described in neuroblastoma only.^7^ In-frame fusions with the 5′ *MLL* or *PAFAH1B2* genes led to overexpression of *FOXR1*.^7^ In accordance with the notion that a constitutively expressed 5′ gene (*RPS25*) might be responsible for the ectopic expression of *FOXR1* in B-cell lymphoma also, *FOXR1* levels were 1000 × higher in the fusion-positive than in the fusion-negative U-2932 subclone. Expression array analyses showed that the *RPS25/FOXR1*-positive U-2932 subclone had the highest *FOXR1* expression level of 55 B lymphoma cell lines tested, three log-scales higher than average (Figure 2a). Quantitative PCR analysis conducted to verify the expression arrays included 17 additional B lymphoma cell lines, revealing that the primary effusion lymphoma cell line CRO-AP3 expressed *FOXR1* at a level similar to the DLBCL cell line U-2932 (Figure 2b). High-density genomic array analysis demonstrated copy-number transition from 3n to 4n in CRO-AP3, occurring 5′ of *FOXR1* (Figure 1c, Supplementary Figure S1B). Quantitative genomic PCR localized the site of amplification to the first 170 bases of exon 1. 5′-RACE, performed to identify potential 5′-mRNA partners in the two *FOXR1* expressing cell lines, confirmed *RPS25* as fusion partner of *FOXR1* in U-2932. In CRO-AP3, the 5′-RACE PCR product terminated inside the amplified region of *FOXR1* exon 1, upstream of the open reading frame. These results suggested that in CRO-AP3, *FOXR1* overexpression was the result of gene amplification without fusion mRNA formation. Fluorescence *in situ* hybridization using a fosmid clone covering *FOXR1* (G248P85736G6) yielded wild-type signals only restricted to chromosome 11 (not shown), leaving the putative 5′ regulatory gene elusive.

Santo *et al.*^7^ reported that *FOXR1* acts as negative regulator of forkhead box factor-mediated transcription and suggested a possible role in tumorigenesis. We found ectopic expression of *FOXR1* in 2/72 (2.8%) B lymphoma cell lines. Both cell lines showed amplification of the *FOXR1* gene. In one cell line, *FOXR1* was fused to a constitutively expressed gene on 11q23, suggesting that the interstitial deletion was responsible for activation of *FOXR1*. Bioinformatic analyses document that the aberrant expression of *FOXR1* is rare, but recurrent in B-cell lymphoma (Figure 2c).^8, 9, 10^

In conclusion, we show for the first time that *FOXR1* fusions, described as candidate oncogenes in neuroblastoma, also occur in B-cell lymphoma. Cell lines U-2932 and CRO-AP3 are presented as models for the functional analysis of *FOXR1*-mediated cellular events.

## Figures and Tables

**Figure 1:**
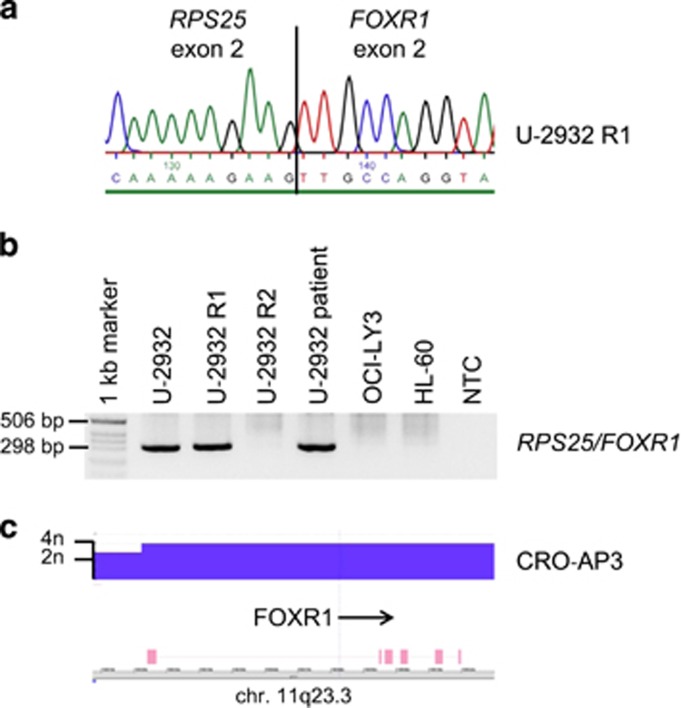
*FOXR1* aberration in B lymphoma cell lines. (**a**) *RPS25* exon 2 / *FOXR1* exon 2 fusion expressed in U-2932 subclone R1. Two additional transcripts targeting *RPS25* exon 2 with *FOXR1* 5′ sequences were also detected. (**b**) *RPS25/FOXR1* fusion in patient's DNA and in one of two subclones of patient-derived cell line. Cell lines OCI-LY3 and HL-60 were used as negative controls. Size of PCR product: 294 bp. (**c**) *FOXR1* was amplified (4n) in CRO-AP3 according to Cytoscan HD Array analysis (Affymetrix, Santa Clara, CA, USA). NTC, nil template control.

**Figure 2:**
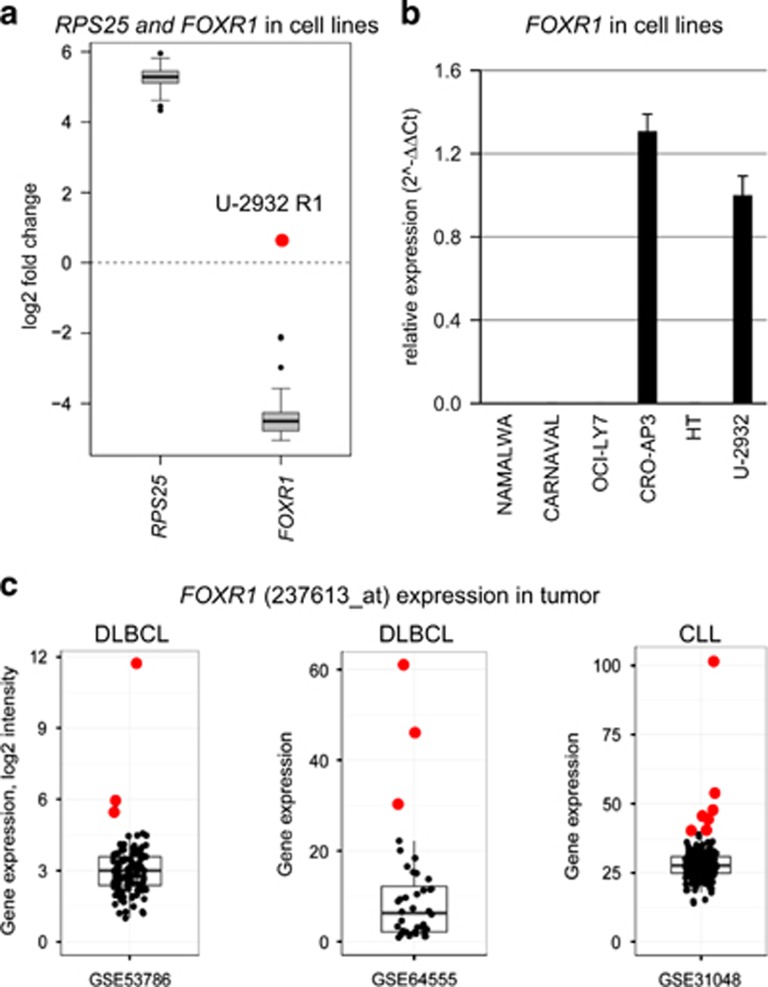
*FOXR1* expression in B lymphoma. (**a**) According to expression array analysis, *RPS25* is constitutively expressed in 55 B lymphoma cell lines, *FOXR1* is highest in the *RPS25/FOXR1*-positive U-2932 subclone (red dot). (**b**) Quantitative reverse-transcriptase PCR-verified ectopic expression in U-2932 subclone R1 and in the PEL cell line CRO-AP3. Cell lines NAMALWA (Burkitt's lymphoma), CARNAVAL, OCI-LY7 and HT (all DLBCL) do not express *FOXR1*. (**c**) Reanalysis of previously published normalized expression profiling data showing ectopic *FOXR1* expression in primary DLBCL and chronic lymphocytic leukemia (processed data from GEO).^8, 9, 10^ Red dots indicate *FOXR1*-high outliers.

## References

[bib1] Zhu Y, Monni O, Franssila K, Elonen E, Vilpo J, Joensuu H et al. Deletions at 11q23 in different lymphoma subtypes. Haematologica 2000; 85: 908–912. 10980627

[bib2] Nagel S, Leich E, Quentmeier H, Meyer C, Kaufmann M, Zaborski M et al. Amplification at 11q23 targets protein kinase SIK2 in diffuse large B-cell lymphoma. Leuk Lymphoma 2010; 51: 881–891. 20367563 10.3109/10428191003699878

[bib3] Salaverria I, Martin-Guerrero I, Wagener R, Kreuz M, Kohler CW, Richter J et al. A recurrent 11q aberration pattern characterizes a subset of MYC-negative high grade B-cell lymphomas resembling Burkitt lymphoma. Blood 2014; 123: 1187–1198. 24398325 10.1182/blood-2013-06-507996 PMC3931189

[bib4] Quentmeier H, Amini RM, Berglund M, Dirks WG, Ehrentraut S, Geffers R et al. U-2932: two clones in one cell line, a tool for the study of clonal evolution. Leukemia 2013; 27: 1155–1164. 23295736 10.1038/leu.2012.358

[bib5] Katoh M, Katoh M. Germ-line mutation of Foxn5 gene in mouse lineage. Int J Mol Med 2004; 14: 463–467. 15289901

[bib6] Schuff M, Rössner A, Donow C, Knöchel W. Temporal and spatial expression patterns of FoxN genes in *Xenopus laevis* embryos. Int J Dev Biol 2006; 50: 429–434. 16525939 10.1387/ijdb.052126ms

[bib7] Santo EE, Ebus ME, Koster J, Schulte JH, Lakeman A, van Sluis P et al. Oncogenic activation of FOXR1 by 11q23 intrachromosomal deletion-fusions in neuroblastoma. Oncogene 2012; 31: 1571–1581. 21860421 10.1038/onc.2011.344

[bib8] Scott DW, Wright GW, Williams PM, Lih CJ, Walsh W, Jaffe ES et al. Determining cell-of–origin subtypes of diffuse large B-cell lymphoma using gene expression in formalin-fixed paraffin-embedded tissue. Blood 2014; 123: 1214–1217. 24398326 10.1182/blood-2013-11-536433 PMC3931191

[bib9] Linton K, Howarth C, Wappett M, Newton G, Lachel C, Iqbal J et al. Microarray gene expression analysis of fixed archival tissue permits molecular classification and identification of potential therapeutic targets in diffuse large B-cell lymphoma. J Mol Diagn 2012; 14: 223–232. 22446084 10.1016/j.jmoldx.2012.01.008

[bib10] Wang L, Shalek AK, Lawrence M, Ding R, Gaublomme JT, Pochet N et al. Somatic mutation as a mechanism of Wnt/β-catenin pathway activation in CLL. Blood 2014; 124: 1089–1098. 24778153 10.1182/blood-2014-01-552067 PMC4133483

