# Supplementary material for: Chromosome 11q23 aberrations activating FOXR1 in B-cell lymphoma
Source: Blood Cancer J. 2016 Jun 10;6(6):e433–. doi: 10.1038/bcj.2016.43 (PMC5141358; doi:10.1038/bcj.2016.43)
Supplement: Supplementary Information [file bcj201643x1.pdf]

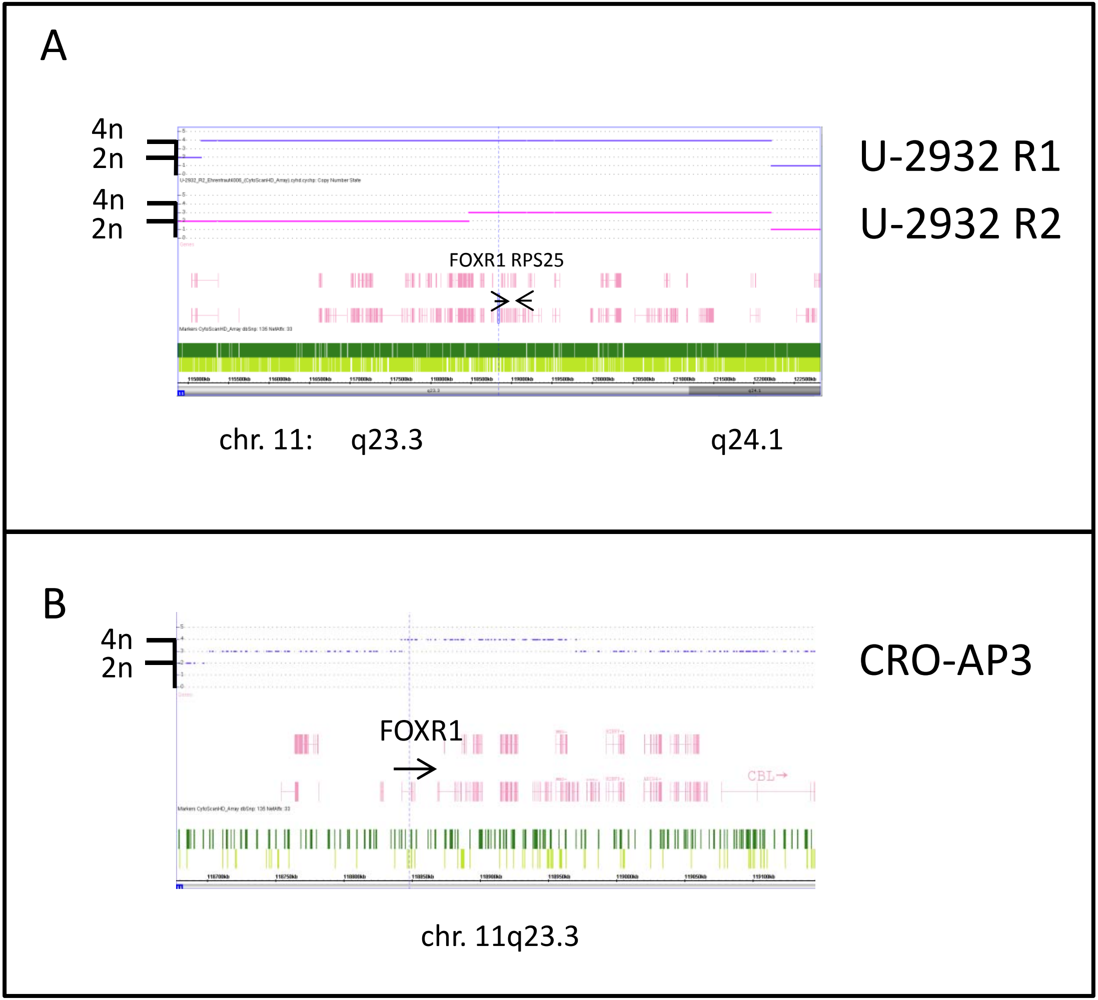
A
4n
2n
4n
2n
U-2932 R1
U-2932 R2
FOXR1 RPS25
chr. 11: q23.3
q24.1
B
4n
2n
CRO-AP3
FOXR1
CBL→
chr. 11q23.3

LEGENDS TO SUPPLEMENTAL FIGURES

Suppl. Figure 1. **Chromosome 11q aberrations.** Chr. 11q23.3 aberrations targeting *FOXR1* in A) cell line U-2932 subclones R1 and R2, and in B) cell line CRO-AP3. Cytoscan HD Array (Affymetrix, Santa Clara, CA, USA) hybridization analysis was performed to identify numerical aberrations. Probes are indicated by green lines, dots show the signal intensity (ploidy status) of the individual probe.
